# Prediction of fluid responsiveness with the LiDCO system

**DOI:** 10.1186/cc9484

**Published:** 2011-03-11

**Authors:** P De Santis, C Marano, F Cavallaro, A Dell'Anna, P De Santis, C Bonarrigo, C Falcone, C Sandroni

**Affiliations:** 1 Catholic University School of Medicine, Rome, Italy

## Introduction

Variation in stroke volume (SV) or related parameters induced by passive leg raising (PLR) measured by several non-invasive methods has been demonstrated to reliably predict fluid responsiveness [1]. The aim of this study was to assess whether variation in SV measured by LiDCO can predict fluid responsiveness in shock states.

## Methods

ICU patients with signs of shock were enrolled. History, clinical information and echocardiogram were obtained. After calibration, hemodynamic evaluation was performed by LiDCO in four subsequent steps: T1 in semi-recumbent position; T2 during PLR; T3 in baseline position; T4 after infusion of 500 ml NaCl 0.9% in 15 minutes. On each step, the heart rate (HR), mean arterial pressure (MAP), absolute and indexed cardiac output and stroke volume (CO/CI, SV/SVI) were measured by LiDCO and the aortic velocity time integral (VTI) by transthoracic echocardiography. Patients whose SVI increased at least 10% after volume load were classified as responders. The ability to predict responder state was assessed for four potential fluid responsiveness indices: variation in SVI, CO, CI and VTI induced by PLR (ΔSVI-PLR, ΔCO-PLR, ΔCI-PLR, ΔVTI-PLR) by means of three statistical methods: comparison (Mann-Whitney) between the mean value of index in responders and nonresponders, correlation (Spearman) between the baseline value of index and increase in SVI after fluids, and the receiver operator characteristic (ROC) curve.

## Results

Fifteen determinations were collected in 13 patients in septic, cardiogenic and hypovolemic shock (males 9/13, age 73.2 ± 5.8, ejection fraction 54% ± 8). Ten patients had spontaneous breathing activity, five had arrhythmias, 11 were under inotropes. The responder rate was 46.7%. Among the studied indices, only ΔSVI-PLR was significantly different in responders and nonresponders (26.9 vs. 1.9, *P *< 0.001). Three indices, ΔSVI-PLR, ΔCO-PLR and ΔCI-PLR, were significantly correlated with increase in SVI after fluids (rho = 0.854 (*P *< 0.001), 0.727 (*P *= 0.002), 0.710 (*P *= 0.003)). ΔSVI-PLR correctly predicted responders state in all cases with a threshold of 9.1%, (sensitivity 100%, specificity 100%, area under the ROC curve (AUC) 1.00 (*P *< 0.001 95% CI = 1.00 to 1.00)). The other indices had values of AUC not significantly different from 0.5.

## Conclusions

The ΔSVI-PLR, measured with the LiDCO system, is a very reliable predictor of fluid responsiveness in a population of ICU patients in shock, including patients with spontaneous breathing activity and arrhythmias.

## References

[B1] Cavallaro Intensive Care Med 2010 36 1475 1483 10.1007/s00134-010-1929-y 20502865

